# Perioperative abnormal electroencephalography in a later-stage elderly with septic shock: a case report

**DOI:** 10.1186/s40981-020-00409-5

**Published:** 2021-01-06

**Authors:** Hirotaka Kinoshita, Tetsuya Kushikata, Daiki Takekawa, Kazuyoshi Hirota

**Affiliations:** Department of Anesthesiology, Hirosaki University Graduate School of Medicine, 5 Zaifu-cho, Hirosaki, 036-8562 Japan

**Keywords:** Electroencephalogram, Sepsis, Sepsis-associated encephalopathy, Ketamine, Postoperative delirium

## Abstract

**Background:**

Patients with sepsis often exhibit abnormal patterns of electroencephalogram (EEG). We report an abnormal EEG pattern in a later-stage elderly patient with septic shock and EEG analysis results.

**Case presentation:**

An 88-year-old woman with bowel perforation underwent emergency Hartmann surgery. On admission to the operating room, she exhibited septic shock. Her bispectral index value was 30 before anesthesia induction, and the EEG displayed slow waves without burst and suppression throughout the surgery. The relative slow-wave ratio [spectral power (0.5–8 Hz)/(0.5–30 Hz)] from anesthetic induction to the end of surgery was 95.1%, whereas the relative alpha frequency [spectral power (8–13 Hz)/(0.5–30 Hz)] was only 2.4%. Although without preoperative neurological abnormalities, she developed postoperative delirium after admission to the intensive care unit.

**Conclusions:**

Intraoperative continuous EEG monitoring in elderly patients with sepsis may be useful to predict sepsis-associated encephalopathy. Therefore, continuous EEG monitoring may improve neurological outcomes.

## Background

Delirium and sepsis are serious complications of critical illness. Sepsis-associated encephalopathy (SAE) is a systemic inflammatory reaction against infection to organs other than the central nervous system. The main symptom of SAE is delirium, and in severe cases, coma. Its diagnosis is based on the evaluation of the sedation and delirium scale after sepsis diagnosis.

Electroencephalogram (EEG) abnormalities, such as a decrease in alpha frequency band power and EEG amplitude, were found in 96% of patients with septic shock [1]. These inhibition degrees of change can reflect cerebral lesion, and changes in EEG may indicate brain damage. Continuous EEG (cEEG) monitoring may be useful to predict SAE, but the EEG of the elderly shows similarly low amplitude, with decreasing alpha waves and increasing slow waves [2].

Here, we report the case of an abnormal EEG pattern in a late elderly patient who suffered from septic shock caused by lower gastrointestinal perforations. We suggest that low-frequency dominant EEG at preinduction may indicate the possibility of SAE.

## Case presentation

We have obtained a written informed consent from the patient and her family for the analysis of the EEG data and publication of this case report.

An 88-year-old woman (height, 150 cm; weight, 40 kg) was conservatively treated for multiple myeloma and diabetes mellitus for approximately 20 years. One day, she suffered from stomachache and subsequently consulted her family doctor. She was diagnosed with sigmoid volvulus and received endoscopic treatment to release the volvulus. She recovered after the treatment, but abdominal pain reoccurred after 3 days. The attending physician diagnosed the patient with bowel perforation caused by the endoscopic treatment and resulting in sepsis. Thus, she underwent emergency Hartmann surgery.

**Fig. 1:**
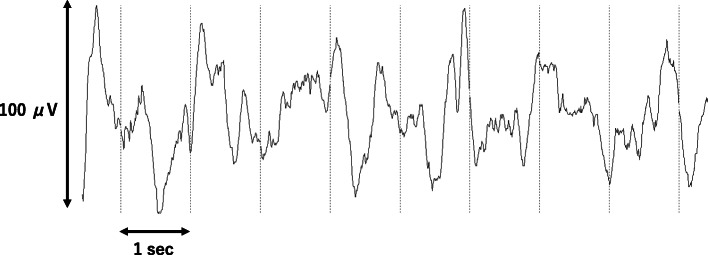
Electroencephalogram before anesthetic induction. This EEG shows lower frequency dominance that is not observed in the healthy elderly

**Fig. 2:**
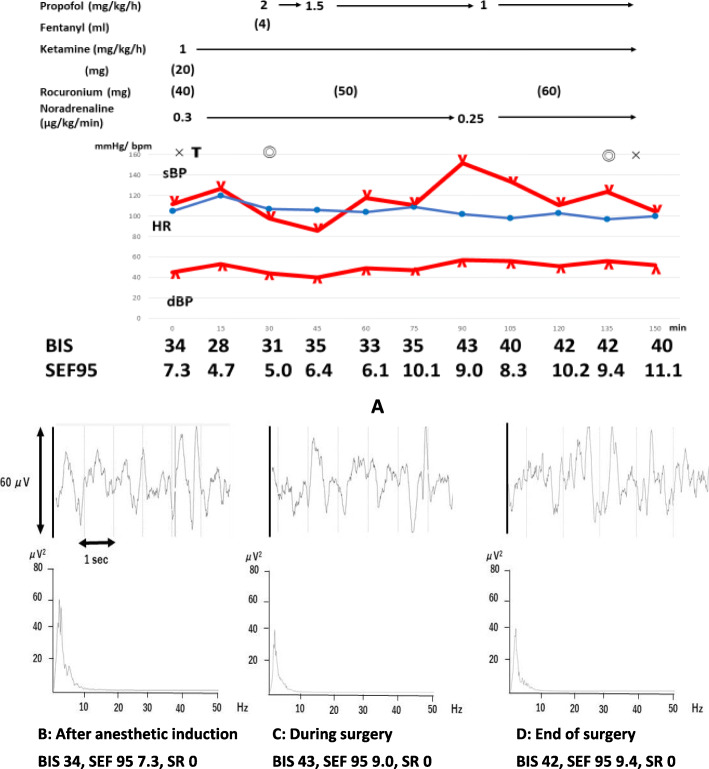
Anesthesia record. EEG waves and frequency analysis at each point. Anesthetic record is shown in (**a**). The BIS value remained from 30 to 40 throughout the perioperative period. Anesthesia was induced by intravenously injecting ketamine (20 mg) and rocuronium bromide (40 mg) at that period. The EEG at lower frequency below 8 Hz was dominant (**b**). The EEG pattern was continuous throughout the surgery (**c**, **d**). Abbreviations: HR, heart rate; sBP, systolic blood pressure; dBP, diastolic blood pressure; BIS, bispectral index; SEF95 (Hz), 95% spectral edge frequency; SR (%), suppression ratio

**Fig. 3:**
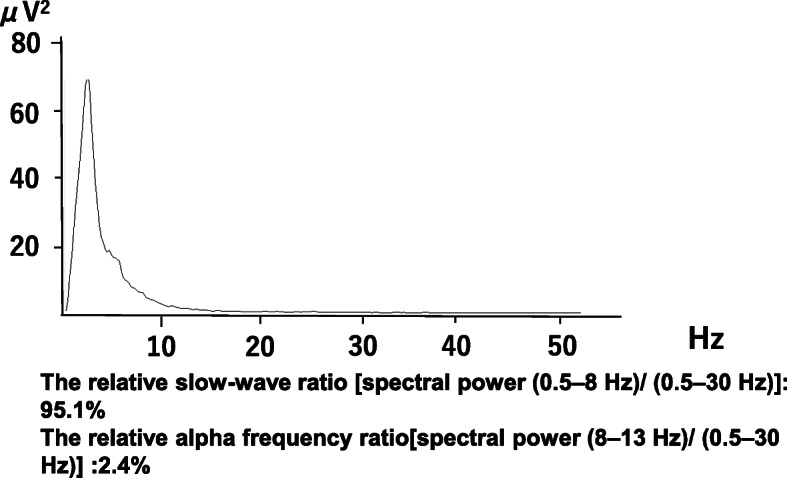
A frequency analysis for the EEG from anesthetic induction to end of surgery. A frequency analysis for the EEG from anesthetic induction to end of surgery is shown. The relative slow-wave ratio [spectral power (0.5–8 Hz)/spectral power (0.5–30 Hz)] from before anesthesia induction to the end of surgery was 95.1%, whereas the relative alpha frequency ratio [spectral power (8–13 Hz)/spectral power (0.5–30 Hz)] was only 2.4%

Upon admission to the operating room, she manifested septic shock; her blood pressure was 90/60 mmHg, and her heart rate was 100 beats per minute, with noradrenaline administration (0.3 μg/kg/min). Nevertheless, verbal communication was possible and the Glasgow Coma Scale was E3V4M6. Furthermore, we monitored the following variables continuously during anesthesia: electrocardiogram, pulse oximetry, end-tidal carbon dioxide concentration, direct arterial blood pressure, central venous pressure, body temperature (esophagus), and urinary output. EEG was monitored and recorded using a BSM monitor ^TM^ (NIHON KOHDEN, Tokyo, Japan) throughout the anesthesia period. Before anesthesia induction, the patient’s bispectral index (BIS) was 30, while her EEG displayed slow waves without burst and suppression (Fig. 1). Anesthesia was induced by intravenously injecting ketamine (20 mg) and rocuronium bromide (40 mg). We used ketamine (1 mg/kg/h), propofol (1–2 mg/kg/h), fentanyl (total dose, 200 μg), and rocuronium bromide to maintain general anesthesia as a total intravenous administration of anesthesia (TIVA). The anesthesia record is shown in Fig. 2a. The EEG waves, 95% spectral edge frequency (SEF95), and suppression ratio (SR) values at anesthetic induction, during surgery, and at the end of the surgery are shown in Fig. 2b–d, respectively. The stored EEG was retrospectively analyzed using the BIMTUS 2 software ^TM^ (KISSEI COMTEC, Tokyo, Japan). We extracted a sample frequency of 128 Hz with the BIMTUS 2 software ^TM^. 3-min EEG frequency analysis at anesthetic induction, during surgery, and at the end of the surgery is shown in Fig. 2b–d, respectively. EEG predominantly consists of slow-wave components without burst and suppression throughout the surgery. The relative slow-wave ratio [spectral power (0.5–8 Hz)/spectral power (0.5–30 Hz)] from before anesthesia induction to the end of surgery was 95.1%, whereas the relative alpha frequency ratio [spectral power (8–13 Hz)/spectral power (0.5–30 Hz)] was only 2.4% (Fig. 3).

Although the patient received minimum amounts of intraoperative anesthetic drugs, she remained immobile and unconscious intraoperatively with BIS values of 30 to 40. She was then admitted to the intensive care unit with tracheal intubation and received ventilator management and continuous hemodiafiltration. After tracheal extubation, she developed postoperative delirium. Nonetheless, her cognitive dysfunction improved as her systemic condition recovered on postoperative day 5.

## Discussion

This case report represents two issues of clinical importance. First, the EEG of this elderly patient with septic shock showed apparent abnormal patterns before anesthesia induction through surgery. Second, the neurological behaviors, such as delirium, of this patient with abnormal EEG may have been induced by perioperative invasion. Therefore, intraoperative cEEG monitoring may predict SAE even if septic shock patients do not have any symptoms.

The BIS of our patient was 30 and EEG displayed slow waves without burst and suppression before anesthesia induction, indicating that she already had SAE at that time. In patients with delirium, routine EEG commonly shows an increased slow-wave activity and a slow and disrupted alpha rhythm [3–6]. Nielsen et al. revealed that delirious episodes in patients with sepsis are associated with the disappearance of high-frequency electrographic cEEG activity and increased in power of low-frequency activity [7]. They also reported the association of EEG and the Confusion Assessment Method for the Intensive Care Unit (CAM-ICU). Normal CAM-ICU scores were associated with continuous or nearly continuous high-frequency cEEG beta activity, preserved cEEG reactivity, and normal cEEG background activity. Conversely, patients with CAM-ICU scores of delirium exhibited a suppressed background cEEG actively. The suppressed cEEG was not observed in any patient without delirium. Although there are reports of patients with sepsis in the ICU [7], there are few reports of EEG during anesthesia in patients with sepsis.

Cerebral rhythmic EEG activity reflects the cortical neurons’ ability to synchronize input from thalamocortical and corticocortical neurons. Generation of high-frequency cortical activity requires fast spiking of high energy–demanding somatostatin-positive interneurons [8].

Ketamine is commonly used for patients with shock. Ketamine, which is an *N*-methyl-d-aspartate antagonist, activates the EEG, despite its sedative effects. Some features of EEG activation by ketamine, such as increases in β-activity, BIS, and SEF95, have already been described [9]. Ketamine also increases the alpha peak frequency, with simultaneous shifting of the bicoherence peak [10]. In this case, such features could not be observed, despite performing TIVA with ketamine as the primary anesthetic.

The abnormal neurological behaviors of this patient may have been induced by not only sepsis but also surgical invasion. Elderly patients with sepsis may be sensitive to anesthetics. An aged brain is significantly vulnerable to immune stimuli compared with a younger brain. Microglia of the aged brain take on a “primed” or “sensitized” phenotype, characterized by dystrophic morphology, progressive accumulation of metabolic stress, increased cell-surface expression of antigen recognition molecules, and an exaggerated inflammatory response to immune challenge [11]. Primed microglia show an increased production of proinflammatory cytokines in response to immune stimulation with a peripheral inflammatory challenge [12]. This patient’s EEG might reflect neuroinflammation. Her neurological behaviors with abnormal EEG may have been induced by surgical invasion and preexisting gastrointestinal perforation itself. Perioperative procedures requiring general anesthesia prolonged neuroinflammation, probably resulting in delirium. The initial processes involved in SAE could lead to cerebral damage [13]; hence, it is important to detect the presence of SAE earlier.

In conclusion, intraoperative cEEG monitoring in elderly patients with sepsis may be useful to predict SAE. The applicability of BIS monitoring for assessing the depth of anesthesia in elderly patients with sepsis should be performed carefully. Predicting SAE from perioperative EEGs may help improve neurological outcomes.

## Data Availability

Please contact the author for data requests.

## Abbreviations

EEG: Electroencephalogram
SAE: Sepsis-associated encephalopathy
cEEG: Continuous electroencephalogram
BIS: Bispectral index
TIVA: Total intravenous administration of anesthesia
SEF95: 95% spectral edge frequency
BSR: Burst and suppression ratio
CAM-ICU: Confusion Assessment Method for the Intensive Care Unit
